# Constructing event graph and mining critical paths of Ebola virus disease: a public health emergency analysis based on WHO disease outbreak news

**DOI:** 10.7189/jogh.16.04341

**Published:** 2026-10-30

**Authors:** Ziran Ni, Yuehong Sun, Guanzhong Feng, Jianxiang Wei

**Affiliations:** 1 School of Management, Nanjing University of Posts and Telecommunications, Nanjing, China; 2 School of Mathematical Sciences, Nanjing Normal University, Nanjing, China; 3 Ministry of Education Key Laboratory of NSLSCS, Nanjing Normal University, Nanjing, China

**Keywords:** event graph, Ebola virus disease, critical path mining, outbreak intelligence, WHO disease outbreak news

## Abstract

**Background:**

Most Ebola virus disease research focuses on clinical diagnosis or static knowledge networks, limiting dynamic spatiotemporal analysis of epidemic evolution. We examined whether an event graph derived from World Health Organization (WHO) Disease Outbreak News (DONs) could reconstruct outbreak evolution across reports and identify response-timing patterns, including delayed interventions, unactioned risk assessments, and cross-border escalation.

**Methods:**

We retrospectively analysed 346 Ebola-related DON reports published through August 2025. We used a large language model-assisted framework to extract events and their relationships. We built an event graph in Neo4j and applied a bounded depth-first search procedure that removed cycles, generated candidate paths, and selected critical paths across six dimensions: case burden, spatial spread, international risk signals, intervention measures, path length, and graph structural importance.

**Results:**

The graph comprised 2233 event nodes and 10,479 relations across 155 event groups; 47 representative critical paths were selected. Laboratory confirmation often marked the shift from uncertain signals to an organised response. Healthcare-facility exposure and community deaths served as sentinel events for infection-control review and transmission-chain investigation. Preparedness, surveillance, and contact tracing generally followed spatial spread and international-risk signals rather than general border restrictions. Repeated burden updates without documented interventions indicated potential response or reporting gaps. Although established control measures aligned with WHO advice, the report defined explicit triggers, deadlines, inter-agency handoffs, and completion indicators less clearly.

**Conclusions:**

The event graph reconstructed longitudinal outbreak and response trajectories that are difficult to capture through descriptive summaries or entity-centred knowledge graphs. Effective Ebola response depends not only on available control measures but also on activating them at appropriate transition points. This framework complements, rather than replaces, WHO guidance, real-time surveillance, and formal epidemiological causal inference.

Ebola virus disease (EVD) is a severe viral haemorrhagic illness first recognised in 1976 and transmitted primarily through direct contact with infectious body fluids [1-5]. Recurrent outbreaks have caused high mortality and substantially disrupted health systems. The 2013–2016 West African outbreak, the largest recorded EVD outbreak, resulted in more than 28,000 reported cases and 11,000 deaths [1,6,7]. Its prolonged, geographically dispersed course shows the need to examine not only epidemic burden but also how transmission events, response measures, and subsequent outcomes connect over time.

Previous EVD research has generated substantial evidence on clinical characteristics, diagnosis, treatment, and prevention [8]. At the population level, Kawuki *et al.* quantified case-fatality patterns across recurrent African outbreaks using meta-analysis [9], while Mbala-Kingebeni *et al.* reconstructed a transmission chain initiated by disease relapse through clinical and genomic investigation [10]. More recently, Consoli *et al.* extracted epidemiological entities and attributes from World Health Organization (WHO) Disease Outbreak News (DONs) to construct a standardised epidemiological knowledge graph [11]. These studies provide valuable evidence on epidemic burden, individual transmission chains, and structured outbreak information. However, aggregate statistics do not retain the sequence of individual events, and case-based investigations generally focus on a specific outbreak or transmission chain. Consequently, they are less suited to reconstructing recurrent event patterns across multiple WHO reports and linking transmission, public health response, and epidemic outcomes within a unified spatiotemporal framework.

Knowledge graphs commonly organise information as entity–relation–entity triples and effectively integrate heterogeneous outbreak facts. Nevertheless, in entity-centred implementations such as epidemiological summary graphs, dates are often represented as attributes rather than as explicit links between events [11,12]. This structure can identify diseases, locations, case counts, and reports, but it does not directly represent whether one event preceded, triggered, or occurred in parallel with another. It also makes it difficult to distinguish repeated mentions of an event and to reconstruct a complete cross-document trajectory linking outbreak emergence, case reporting, risk assessment, intervention, and epidemic termination. These limitations stem from the static, entity-centred implementation used for outbreak summarisation, not from an inherent inability of all knowledge graphs to incorporate temporal information.

An event graph provides an event-centred representation in which events, not just entities, are modelled as nodes, and their temporal, causal, and other semantic associations are encoded as edges [13–15]. By retaining each event's time, location, participants, and action, this representation enables fragmented statements from multiple reports to be organised into ordered, traversable event trajectories. It is therefore suitable for examining how EVD transmission events and public health interventions evolve and interact across reports. Accordingly, we aimed to extract six categories of EVD-related events and their causal, sequential, and parallel relationships from WHO DONs. We also aimed to construct an EVD event graph that explicitly represents these event-level associations, and mine critical paths to characterise recurrent transmission and response patterns. We thereby evaluated the utility of an event graph for organising longitudinal outbreak reports and identifying epidemiologically relevant event chains.

Accordingly, this study was guided by the following research question: Can an event graph constructed from WHO DONs reconstruct cross-report Ebola outbreak evolution and identify response-timing relationships that are not readily visible in conventional descriptive summaries? Specifically, we examined whether the event graph could: consolidate repeated WHO updates into outbreak-level event chains; identify critical paths involving delayed intervention, cross-border or cross-regional escalation, risk reassessment, and community-death-related tracing; and translate these path patterns into graph-based warning rules for retrospective outbreak intelligence.

## METHODS

### Data sources

We used an observational study design, including a secondary (retrospective) data analysis. We sourced the data from the WHO Emergency Events Webpage's DONs. The WHO system compiles and continuously updates this data source [16,17], which includes records of various public health emergencies globally, including infectious disease outbreaks, conflicts, and natural disasters. It serves as an authoritative and reliable resource for public health analysis. Each report includes essential information, such as the nature of the emergency, the affected country or region, the incident report date, the degree of health hazard, and the response measures implemented by the WHO. The transparency and accessibility of the data provide a robust foundation for this study. We used web crawler technology for data collection. Initially, we collected all reports of public health events related to virus outbreaks on the WHO emergency event page, then focused on the Ebola virus outbreak for topic screening. Ultimately, we obtained 346 effective report texts as the foundational data sources for subsequent information extraction.

We included all reports of human EVD outbreaks published on the WHO emergency website through August 2025. We excluded reports of infections not caused by Ebola virus, animal infection events without documented human transmission, and duplicate reports with identical or substantially overlapping content.

The reports share a similar structure, primarily comprising the following sections: situation description, detailed description, public health response, WHO risk assessment, epidemiology, and further information. The initial report's content module is relatively straightforward, whereas the subsequent reports are more comprehensive. During data preprocessing, we uniformly removed the ‘supplementary information’ section from all reports, as it contained only links to other information, had no analytical value, and did not substantively affect subsequent research. Concurrently, we removed redundant blank lines from the reports, retaining only core analytical content to ensure data consistency and validity.

### Information extraction

#### The concept of event and relationship

In the theoretical framework of event graph research, an event is defined as an objective state change or factual occurrence triggered by one or more agents through a specific action or process within a defined spatiotemporal scope [18,19]. Its core features are semantic integrity and structured expression, the basic semantic units that support logical relationship modelling between events. From a formal perspective, an event can be represented as a multi-tuple form: *E* = (*e_id*, *T*, *S*, *A*, *P*, *Attr*), where *e_id* is the unique identifier of the event, *T* is the time attribute (including the start time *t_start* and the end time *t_end*), *S* is the spatial attribute (including the geographical scope and hierarchy), *A* is the set of participants (*A* = *{a_1, a_2, ..., a_n}*; each participant is associated with a specific semantic role), *P* is the description of the core action or state change, *Attr* is the set of event attributes (including key features such as event type, polarity, and tense). This definition not only continues the classical definition of the core elements of events in the field of event semantic modelling [20], but also realises the structured encapsulation of semantic information through the multi-group form, which can accurately describe the dynamic characteristics and composition logic of events in the fields of public health and disaster emergency. It provides a unified semantic basis for extracting and modelling causal, temporal, and conditional relationships between events in the event graph and ensures systematic representation of event-association logic.

Combining the characteristics of unstructured text event extraction and the requirements of event graph construction, the public health event defined in this research is: in a specific domain context, the semantic unit activated by the trigger word can reflect the core dynamic facts, and the formal representation still uses the multivariate group *E* = *(e_id, T, S, A, P, Attr)*, but it is necessary to clarify the difference between this definition and the traditional standard event definition. Because event information is derived directly from unstructured text, it is affected by ambiguous descriptions and missing information. The extracted event does not need to contain all tuple elements. The core only needs to retain the core action/process *P* corresponding to the trigger word and the key semantic elements to ensure that the event has distinguishability and relationship modelling capabilities. This definition not only preserves events' essential characteristics as dynamic semantic units, but also adapts to the practical needs of unstructured text information extraction, providing a flexible, rigorous basis for constructing event correlations in the event graph.

Based on the definition of the above events, we also explain the relationship between events. The relationship between events is roughly divided into three categories:

1. Causality: refers to the cause-effect association between event E1 and E2 [21,22], wherein E1 directly affects the probability of E2 occurring by changing the preconditions of the event, rather than the two events merely co-occurring in time. The tangible manifestation of this relationship is an action or information-flow chain that connects E1 and E2, such that changes to E1 affect the likelihood of E2 occurring. In text and natural language processing, causality is expressed through a cause-effect link, requiring that E1 precede and influence E2.

2. Sequential: events follow a temporal sequence [23]. E1 occurs before E2; however, this does not imply that E1 causally influences the emergence of E2. Rather, it represents a purely temporal association distinct from causality. This relationship is classified into two forms: strict succession (wherein one event initiates immediately upon the termination of another) and loose succession. Its fundamental characteristic is temporal sequentiality, in which one event occurs after another.

3. Parallel: indicates that there is neither a causal relationship nor a dependent sequential step between events that typically occur simultaneously or exist as independent branches of the same process [24]. Although such events may share overarching contexts or common causes, they do not influence one another. In the analysis, these events can be associated with a ‘relational event history’ or parallel sequences, but they do not demonstrate a clear causal direction.

#### Event extraction

Technological evolution in the field of natural language processing has shown that the language representation ability constructed by large language models (LLMs) through self-supervised learning has achieved state-of-the-art performance in natural language understanding and natural language generation tasks [25], which can effectively solve the problem of accurately extracting key information in unstructured epidemic texts. This study employs DeepSeek-R1 – a large language model with enhanced reasoning capabilities via the chain-of-thought approach – as the core tool for event and relation extraction thought [26,27] (Appendix S1 in the **Online Supplementary Document**). The chain-of-thought mechanism further improves the model's ability to infer logical relationships in complex events by simulating humans' gradual reasoning process and provides reliable support for event classification and relationship determination [28]. The event extraction workflow comprised text cleaning, event extraction, temporal ordering, entity standardisation, and format unification (**Figure 1**, Panel A).

**Figure 1:**
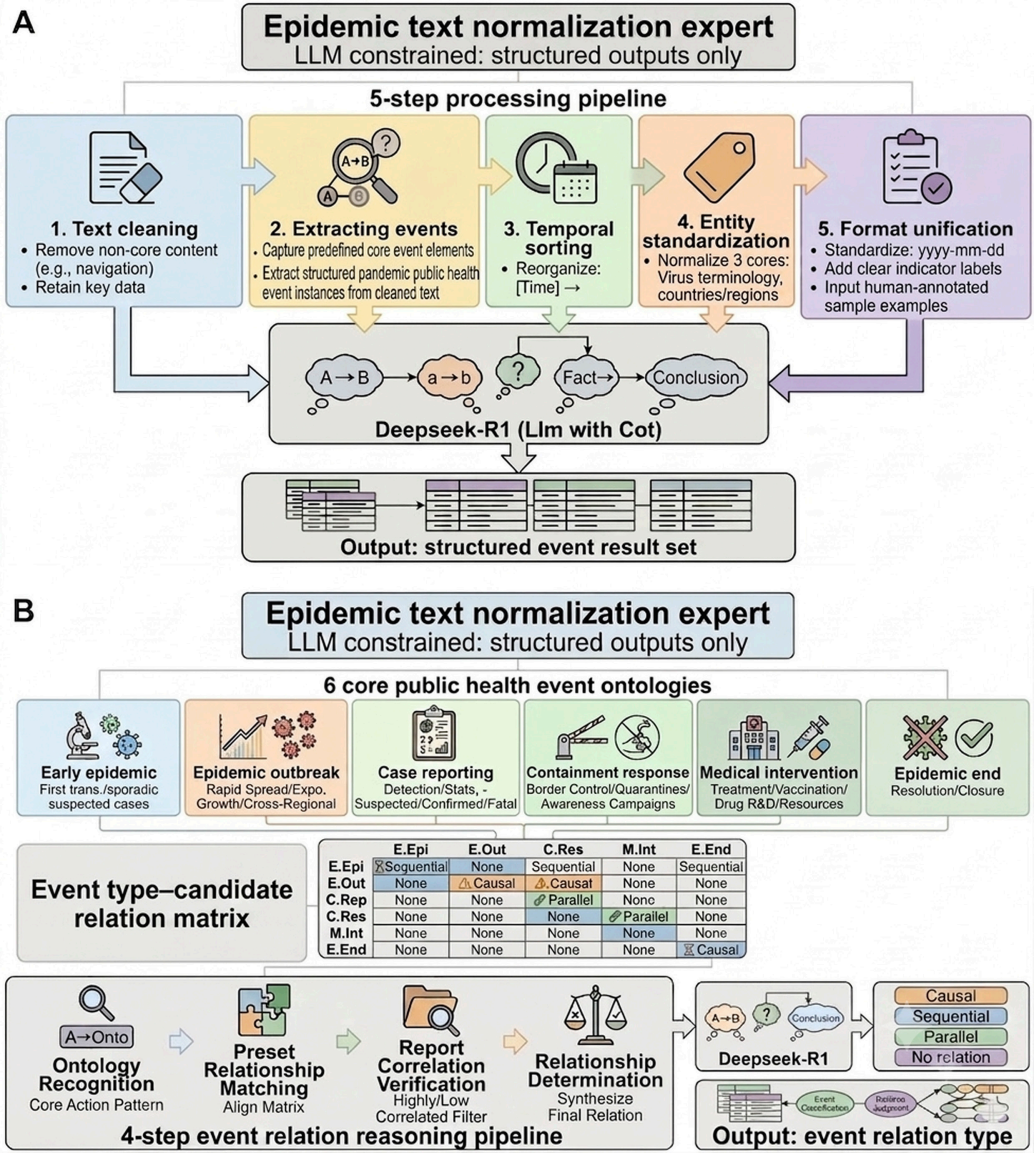
Technical frameworks for event and event-relation extraction. **Panel A.** Event extraction framework. **Panel B.** Event-relation extraction framework. CoT – chain-of-thought, LLM – large language model.

The framework initially establishes the model as an 'epidemic text normalisation expert,' constraining the output to structured results without requiring additional analysis or interpretation and adhering strictly to the predefined example format. The first step involves text cleaning, which removes non-essential web navigation content and keeps only the first occurrence of duplicate data. The second step extracts events from the text based on the event's defined elements. The third step organises the event sequence using temporal markers such as dates and ‘epidemic X week’ as anchor points, and restructures the text into the ‘time: event content’ format to prevent word-order confusion. The next step is entity alignment, which standardises the three core entity names, institutions, virus terms, and countries/regions, using predefined mapping rules. The next step unifies the format and converts the time to the ‘YYYY-MM-DD’ standard format. Finally, the process concludes with event element extraction, identifying and extracting all recognisable components defined by the multi-tuple representation *E* = (*e_id*, *T*, *S*, *A*, *P*, *Attr)* to the fullest extent possible. The extracted elements encompass temporal attributes (*T*), spatial attributes (*S*), participant sets (*A*), core action descriptions (*P*), and supplementary attributes (*Attr*) including event type, polarity, and tense. Given the ambiguous and incomplete nature of unstructured epidemic text, we do not artificially supplement tuple elements absent from the original content; we retain only verifiable semantic elements to ensure event distinguishability and enable subsequent event relation modelling. To provide in-context guidance for event extraction, we manually annotated five WHO DONs reports and incorporated them into the prompt as few-shot examples. These examples illustrated the expected event boundaries, temporal normalisation, entity standardisation, and structured output format. We did not update any model parameters; the annotated examples served solely as prompt-based guidance to improve the consistency of model outputs (Appendix S2 in the **Online Supplementary Document**).

#### Event relation extraction

Considering the event set results, we also need to ascertain the interrelationships among these events. Event-relation extraction combines event-type recognition, candidate-relation matching, report-level association verification, and final relation determination (**Figure 1**, Panel B).

First, to enable a more comprehensive examination of the outbreak's critical path, we categorised epidemic events into six distinct types. These categories are delineated as:

− Early epidemic events: occurring in the early stage of an epidemic, characterised by the emergence of signs of human-to-human Ebola virus transmission, the reporting of sporadic suspected cases in local areas, and no identified clear transmission chain or large-scale community transmission.
− Epidemic outbreak events: rapid expansion of the scope of virus transmission, exponential growth of the number of infected people beyond the local prevention and control carrying capacity, and the formation of a cross-regional transmission trend of related events.
− Case report events: relevant events of the discovery, registration, statistics, and publicity of suspected cases, confirmed cases, and deaths of Ebola by medical and health institutions, disease control departments, or relevant organisations.
− Epidemic response events: related to prevention and control measures, such as entry and exit control, epidemic area blockade, personnel isolation, and public health publicity implemented by governments and international organisations in response to the Ebola epidemic.
− Medical intervention events: medical-related events such as clinical treatment, vaccination, drug development and application, and medical resource allocation for Ebola-infected patients.
− Epidemic termination events: denote the formal declaration of the conclusion of the epidemic.

To improve the LLM's ability to discern event relationships and support consistent relation extraction, we constructed an empirical event type–candidate relation matrix based on repeated observations of event sequences in WHO DON reports and preliminary extraction results. This matrix was not intended to represent a formal ontology or expert consensus. Instead, it served as a heuristic guide to narrow the plausible candidate relations between ordered event-type pairs. The matrix alone did not determine the final relation label; local textual evidence, temporal order, and the contextual meaning of the two events did. This matrix uses the six event-type combinations above and posits Sequential, Causality, and Parallel relationships between them (**Table 1**). The matrix guides the LLM to combine event-type and contextual semantics when determining event relationships, improving the rationality of relation extraction.

**Table 1:** Event type–candidate relation matrix

Combinations of event types	Sequential relationship	Causality relationship	Parallel relationship
Early epidemic events→ epidemic outbreak events	Initial→outbreak (time successively)	Initially lead to an outbreak (causality)	
Epidemic outbreak events → case report events	Outbreak → report (time successively)	Outbreak pushes report (causality)	
Epidemic outbreak events → Epidemic response events	Outbreak → response (time successively)	Outbreak triggers response (causality)	Parallel occurrence (parallel)
Epidemic outbreak events → medical intervention events	Outbreak → intervention (time successively)	Outbreak pushes intervention (causality)	
Case report events→ epidemic response events	Report → Response (time successively)	Data drives response (causality)	Parallel occurrence (parallel)
Case report events → epidemic termination events	Report → End (time successively)	Ended with data proof (causality)	
Epidemic response events → medical intervention events	There is compliance (response → intervention) in some scenes		Parallel occurrence (parallel)
Epidemic response events → epidemic termination events	Response → end (time successively)	Response pushed to end (causality)	
Medical intervention events → epidemic termination events	Intervention → end (time successively)	Intervention drives the end (cause and effect)	

Subsequently, the LLM (DeepSeek-R1) uses a four-step reasoning framework for relation extraction. It must accurately identify four types of associations between events: ‘causality, sequential, parallel, and unrelated’. The initial step involves event type recognition, which is achieved through ‘core action + occurrence stage’ feature matching to clarify the event type. The second step entails correlation verification. If the two events originate from the same report, they are deemed highly correlated, and the predefined relationship is preferentially referenced and assessed. Otherwise, in the absence of a clear correlation clue, the events are initially considered to have 'low correlation'. The third step involves predefined relationship matching, where inference rules are constructed based on event-type combination logic, and decisions are derived by querying the corresponding event type–candidate relation matrix. The fourth step involves relationship determination. By synthesising the outcomes of the preceding three steps, the model identifies the final relationship type and outputs only the relationship category. To provide in-context guidance for relation classification, we incorporated ten manually annotated event pairs into the prompt as few-shot examples, allowing the model to capture implicit association rules among epidemic events (Appendix S3 in the **Online Supplementary Document**).

#### Validation of model-based information extraction

To evaluate the reliability of LLM-based information extraction in epidemic event analysis, we performed double-annotator manual validation for both event extraction and event relation extraction tasks. We randomly selected 20 WHO DON reports and 100 extracted event pairs from the overall experimental results for validation.

For event extraction validation, two annotators independently annotated the 20 selected reports. We then compared the two sets of manual annotations. We defined the two experts' consistent event extraction results as the standard ground truth. For inconsistent annotations, the two experts held group discussions to resolve discrepancies and reach a unified conclusion, forming the final benchmark data set. We then compared the finalised manual standard results with the LLM's event extraction outputs to evaluate model performance.

Similarly, for event relation extraction validation, the two experts independently assessed 100 randomly selected event pairs. The experts judged the relation types of event pairs (including Causality, Sequential, Parallel and Unrelated) based on textual context, event temporal order, and epidemiological logic. We adopted consistent relational annotations as standard answers, and unified discrepant judgments through expert negotiation to establish the final ground truth. We further compared the unified manual results with the relational classification results generated by the LLM.

For evaluation, we considered an LLM-extracted event correct when its core semantic information matched the manually calibrated standard event, allowing minor differences in literal expression; we considered an LLM-extracted event relation correct when its label matched the expert-consensus ground truth. On this basis, we calculated precision, recall, and F1-score to quantify event-extraction performance, and we used classification accuracy to evaluate the effect of event-relation extraction (Appendix S4 in the **Online Supplementary Document**).

### Construction of the Ebola event graph

After event and relation extraction, we constructed an initial fine-grained Ebola event graph from individual WHO DON reports. Because WHO reports are generally published as successive updates, different stages of the same outbreak often span multiple report-level event clusters. Using these fragmented clusters directly would make it difficult to reconstruct continuous outbreak-evolution paths. Therefore, before critical path mining, this study consolidated large outbreak events. This step merged fine-grained report-level event clusters that belonged to the same real-world outbreak. It was based on manual review and judgment. Specifically, we manually checked the original WHO reports and related outbreak information, including outbreak country or region, event time, virus type, affected health zone, case and death descriptions, report update sequence, and semantic continuity between events. We merged clusters only when the available evidence supported their belonging to the same outbreak process; otherwise, we retained them as separate event groups. This procedure organised all 2233 event nodes into 155 outbreak-related event groups.

After outbreak-level consolidation, we removed duplicate events within each consolidated group because the same case update, death report, laboratory confirmation, contact-tracing activity, vaccination measure, or risk assessment could be repeatedly described in successive WHO reports. Retaining these repeated descriptions as separate nodes would overrepresent frequently reported information and affect subsequent path evaluation. We identified duplicate or highly overlapping events by manually comparing their content, time, location, subject, and public-health meaning. When multiple nodes described the same epidemiological event, we retained the most complete and standardised representation and merged redundant descriptions. We reviewed relations involving merged events alongside the corresponding event content and retained, adjusted, or supplemented them when supported by temporal order, epidemiological logic, and report context. This outbreak-level graph reduced repeated reporting and cross-report fragmentation, providing a structured basis for subsequent temporal correction, cycle removal, and critical path mining.

### Critical path mining

To identify representative epidemic-evolution chains from the Ebola event graph, the critical path mining procedure comprised two stages. First, we performed temporal correction and cycle removal on the causal-sequential subgraph to obtain an acyclic directed graph. Second, bounded depth-first search (DFS) [29] was used to generate candidate paths within each outbreak-related event group. We evaluated paths with at least six event nodes using six dimensions: case burden, spatial spread, international-risk signal, intervention measure, path length, and structural importance. We min–max normalised the six-dimension values and summed them with equal contribution to obtain a composite path score. For each event group that produced eligible candidate paths, the path with the highest composite score was retained as its final critical path.

#### Cycle correction

Before generating candidate paths, we retained only Causality and Sequential relations as directional edges. We preserved parallel relations in the complete event graph but excluded them from path mining because they did not indicate a direction of outbreak evolution. We then performed cycle correction in two stages. First, we checked relation directions against the available event dates. We reoriented clearly backward sequential relations, whereas we removed clearly backward Causality relations because reversing them would alter their inferred dependency meaning. Relations involving identical dates, overlapping time ranges, or unresolved dates retained their original directions and were treated as temporally uncertain. Stable event identifiers were not used as evidence of temporal order. After temporal correction, Tarjan’s strongly connected component algorithm [30] was applied to detect residual directed cycles. Only one cyclic component containing three event nodes was identified. Because the number of cycles was very small, we manually reviewed the event descriptions, temporal order, and relation semantics within this component. After this review, we removed one Sequential relation to break the cycle. We then repeated the Tarjan analysis, confirming that no cyclic components remained. The resulting acyclic causal-sequential graph was used to generate candidate paths and evaluate them.

#### Candidate-path evaluation

After correcting relations and removing cycles from the constructed event graph, we generate candidate paths within each outbreak-related event group based on the calibrated causal and sequential relations. A candidate path *P_i_* is defined as an ordered sequence of event nodes connected by relational edges, denoted as *P_i_* = *(v_1_,v_2_,…,v_k_)*. To eliminate short chains with limited information value, we retain only paths with at least six event nodes, satisfying *N(P_i_)*≥6, where *N(P_i_)* denotes the total count of event nodes in path *P_i_*. Candidate paths were generated using bounded DFS within each connected event-group component. Because the number of simple paths can increase exponentially with graph depth and branching, unrestricted path enumeration may become computationally infeasible in dense graphs. To control the search space, we set the maximum path length to 30 nodes, examined no more than 35 successors per node, and generated no more than 6000 candidate paths per component. Therefore, the practical computational cost depended on how many search states were explored within these predefined bounds. These constraints kept the path-mining procedure computationally manageable while preserving sufficiently informative candidate paths for subsequent evaluation. We quantitatively evaluate the retained paths across six dimensions: case burden, spatial spread, international risk signal, intervention measure, path length, and graph structural importance, forming the evaluation dimension set *D* = *{B, S, I, M, L, Q}*. The calculation specification for each dimension is elaborated as follows:

Case burden score (B) characterises epidemic severity along a path by taking the maximum case count across all nodes in the path. Epidemic burden data typically exhibit a strongly right-skewed distribution, with values spanning multiple orders of magnitude from sporadic infections to large-scale outbreaks. Direct adoption of raw values would cause high-burden nodes to dominate the scoring excessively. We therefore apply a logarithmic transformation to compress the scale and smooth the impact of extreme values, enabling more comparable scores across epidemic scales. The ‘+1’ term in the formula avoids invalid logarithmic operations when the burden value equals zero. The formula is expressed as:



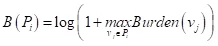



The spatial spread score (S) is scored hierarchically based on cross-regional transmission signals embedded in the path: cross-border spread receives a score of 1, domestic cross-regional spread a score of 0.5, and the absence of spatial spread signals receives a score of 0. The final score of a path is the maximum score among all its constituent nodes:



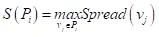



International risk signal score (I) is a binary indicator that identifies whether a path contains official WHO risk escalation signals. The score takes the value of one if the path includes WHO risk-upgrading events, and 0 otherwise:



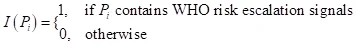



Intervention measure score (M) evaluates coverage of five predefined intervention-measure categories in each path: isolation, contact tracing, vaccination, safe burial, and surveillance and screening. We set the denominator to five because these five categories constituted the complete intervention-measure set used in this study. The score equals the number of distinct measures contained in a path divided by five. For example, a path containing three measures receives a score of 3/5.



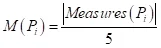



where *|Measures(P_i_)|* denotes the number of these categories.

The path length score (L) is normalised against the maximum node count of all candidate paths within the same event group, reflecting the path's event-coverage completeness. Longer event chains generally carry more complete outbreak evolution information; hence, we incorporate this dimension into the evaluation framework. Nevertheless, it serves as only one of multiple dimensions rather than the sole selection criterion, to avoid biased preference for excessively long paths:

*L*(*P_i_*) = *N*(*P_i_*)

Graph structural importance score (Q) measures the positional importance of events in the corrected causal-sequential graph using two node-level structural indicators: betweenness centrality [31] and descendant reach [32]. Betweenness centrality *BC(v)* quantifies the extent to which an event node acts as a bridge across different stages of outbreak evolution, calculated as the proportion of all pairwise shortest paths that pass through node *v*:



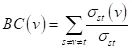



where *σ_st_* is the number of shortest paths from node *s* to node *t*, and *σ_st_ (v)* is the count of such paths traversing node *v*.

Descendant reach (*DR(v)*) measures the downstream influence scope of an event, defined as the number of nodes reachable from node v *via* causality or sequential relations:



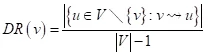



where *v⇝u* indicates that node *u* is reachable from node *v* through causality or sequential edges. *|V|* denotes the number of nodes contained in the entire cluster.

Since *BC(v)* and *DR(v)* have different scales, we normalise each indicator independently using min-max normalisation, where *BC(v)* denotes the normalised betweenness centrality, and *DR(v)* downstream influence of node v. The node-level structural score is then computed as an equally weighted combination of the two normalised indicators: *Q*(*v*) = 0.5*BC(v)* + 0.5*DR(v)*. This score captures both the bridging role and the downstream influence of each event node.

For each candidate path *P_i_*, let *k* denote the number of nodes in *P_i_*. The path-level structural importance score is defined as the average structural score of all nodes in the path:



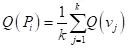



Using the average value instead of the aggregated total prevents longer paths from being favoured merely for containing more nodes, thus reducing the confounding effect of path length on structural ranking.

#### Candidate path selection

Given the inconsistent value ranges across the six evaluation dimensions, we min-max normalise each dimension within the candidate path set for the corresponding event group. For candidate path *P_i_*, its normalised value on dimension *j* is calculated as:



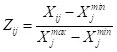



where *X_ij_* denotes the original score of path *P_i_* on dimension *j*, and 0≤*Z_ij_*≤1, represents the corresponding normalised score. If all candidate paths in an event group share the same value on a given dimension, set its normalised value to zero, as that dimension provides no discriminative power for path differentiation. The composite score of candidate path *P_i_*, denoted as *S_i_*, is derived by summing the normalised scores across all six dimensions:



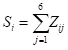



For each event group *g*, we first identify all eligible candidate paths within that group. Each path is assigned a composite score by summing its normalised values across the six evaluation dimensions. The path achieving the highest composite score is then selected as the critical path for event group *g*.

## RESULTS

### Descriptive statistical results

We performed a descriptive statistical analysis on the extracted data. This research manually summarised the 346 Ebola-related DONs reports by country. Reports were unevenly distributed, with the Democratic Republic of the Congo contributing the most (**Figure 2**).

**Figure 2:**
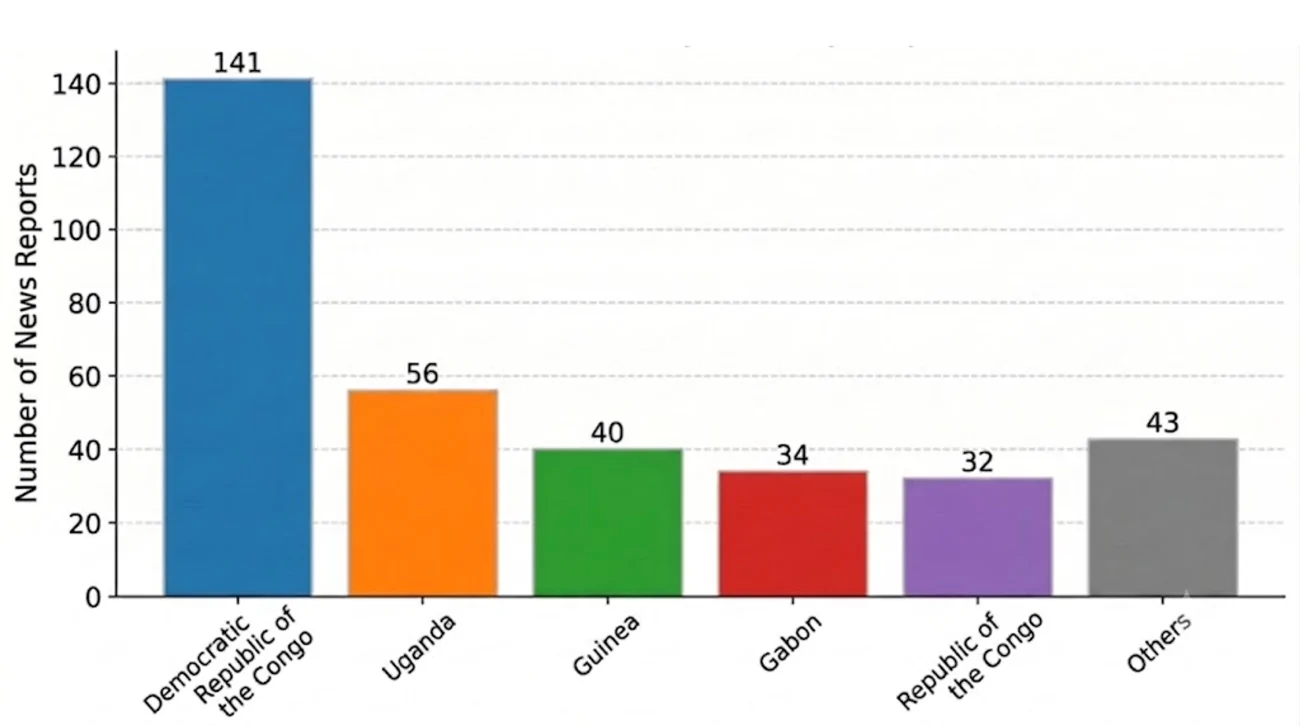
Number of Ebola-related DON reports by country. DON – Disease Outbreak News.

In this study, among the 346 EVD reports, the outbreak's spatial distribution showed significant regional clustering and high-frequency activity. The Democratic Republic of the Congo was the most frequently represented country in the corpus, accounting for 141 of the 346 reports (40.8%). These findings align closely with the long-term monitoring conclusions of the WHO and the United States Centers for Disease Control and Prevention. Uganda ranked second with 56 outbreak-related reports, most of which involved Sudan ebolavirus outbreaks. Historical regional epidemics underscore East Africa's risk profile as a hotspot for cross-species virus transmission. Guinea and Gabon ranked third and fourth, with 40 and 34 reports, respectively.

### Results of information extraction

The LLM-assisted extraction procedure generated structured event records containing event descriptions, event dates, and corresponding source reports (Table S1 in the **Online Supplementary Document**). Based on these records, representative event-pair outputs illustrate how the model assigned relation labels according to the temporal and contextual associations between events (Table S2 in the **Online Supplementary Document**).

To evaluate the reliability of the model-based information extraction, this study manually validated a random sample of 20 WHO DON reports and 100 event pairs. For event extraction, the two reviewers manually identified 132 event units from the selected reports. The LLM extracted 118 event units from the same reports, of which 94 were judged to be consistent with the manually identified events. The event extraction precision was 79.66%, recall was 71.21%, and F1 was 75.20%. For event relation extraction, we randomly selected 100 event pairs and manually reviewed them. Of these, 78 relation labels assigned by the model matched manual judgment, yielding an overall relation classification accuracy of 78.00% (**Table 2**). The model performed relatively well in identifying Sequential relations, while most errors occurred in distinguishing Causality from Sequential relations. This confusion was particularly common when the report described a response action following an outbreak update, but did not explicitly state a causality connection.

**Table 2:** Manual validation results of model-based information extraction

				Performance, %			
**Validation task**	**Manual sample size**	**Model output**	**Correct/matched results**	**Precision**	**Recall**	**F1**	**Accuracy**
Event extraction	132 manually identified events from 20 reports	118 extracted events	94 matched events	79.66	71.21	75.20	
Relation extraction	100 event pairs	100 model-assigned relations	78 correct relation labels				78.00

F1 – harmonic mean of precision and recall

Overall, the validation results indicate that the LLM-assisted extraction method provided a usable but imperfect structured basis for constructing the Ebola event graph. The model captured most major public health events, but omissions and boundary inconsistencies remained in complex report paragraphs. Therefore, we needed to consolidate outbreak-level events, remove duplicates, correct relations, and remove cycles before critical path mining.

### Results of graph construction

A partial view of the constructed event graph illustrates the temporal organisation of event nodes and the causal, sequential, and parallel relations among them (**Figure 3**). Before critical path mining, the initial relation data set contained 8919 records. We added 1749 new relations generated during event cluster consolidation, bringing the total to 10,668 relational records. Two records with blank relation labels were excluded, leaving 10,666 typed relations. After temporal correction and cycle removal, the final graph contained 10,479 relations: 4075 Causality, 3707 Sequential, and 2697 Parallel. We assigned all 2233 event nodes to 155 event groups.

**Figure 3:**
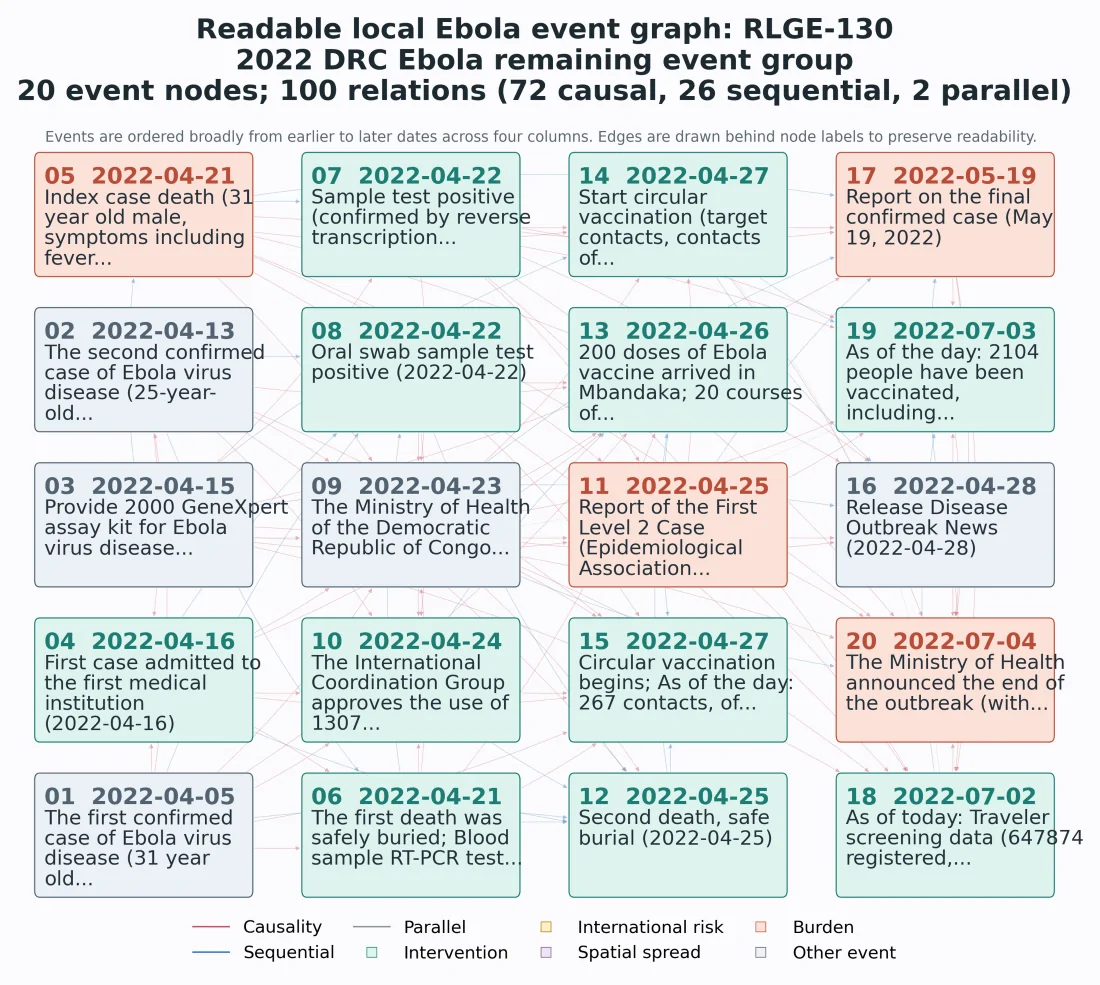
Partial view of the constructed Ebola event graph for a representative event group. The graph contains 20 event nodes and 100 relations, comprising 72 causal, 26 sequential, and two parallel relations. DRC – Democratic Republic of the Congo, RT-PCR – reverse transcription polymerase chain reaction.

The path-mining graph contained 2209 nodes from 153 groups and 7724 within-group directed relations, including 4051 Causality and 3673 Sequential relations. The node count is 2209 because we excluded isolated nodes, nodes from groups removed during cleaning, and nodes not connected by valid within-group causality or sequential relations from the path-mining graph.

We examined all 153 event groups with eligible directional relations, of which 47 generated at least one candidate path with at least six nodes. Bounded DFS generated 72,357 eligible candidate paths. After the six evaluation dimensions were min–max normalised and summed, the highest-scoring path was selected from each of these 47 event groups, producing 47 final critical paths (**Table 3**).

**Table 3:** Examples of the final critical paths

Path ID	Critical path ID	Event group	Critical path content
1	PEKP-REP-001-RLGE-122	RLGE-122	Report a confirmed case hospitalised after staying in the community for 4 days and infecting two people → Five newly confirmed cases were reported in Beni Health District; all were known contacts but had not been followed in time → Beni became an epidemic hotspot, accounting for 64% of cases reported during the preceding 21 days → Mambasa and Butembo reported prolonged periods without new cases → Cumulative burden reached 3421 cases and 2242 deaths → Contact tracking and alert investigation improved → WHO assessed national and regional risks as very high and global risk as low → WHO recommended against travel and trade restrictions on the Democratic Republic of the Congo.
2	PEKP-REP-002-LGE-012	LGE-012	A total of 713 cases and 439 deaths were reported, including 61 infections among medical staff; WHO assessed national and regional risks as very high → The burden increased to 2025 cases and 1357 deaths, with continued transmission in multiple health zones → Regional and national rapid-response teams were mobilised → WHO maintained a very high or extremely high national and regional risk assessment → Confirmed cases and deaths were reported in Goma and Nyiragongo → A total of 232 contacts, including 114 high-risk contacts, were monitored → Mambasa became a new hotspot → Nyiragongo reported no new confirmed cases for 21 days, and all contacts completed follow-up → The cumulative burden reached 2997 cases and 1998 deaths and later 3175 cases and 2122 deaths → WHO reported the need for additional response funding.
3	PEKP-REP-003-LGE-010	LGE-010	A total of 1711 cases and 932 deaths were reported across Guinea, Liberia, Nigeria and Sierra Leone → WHO convened Member States to discuss outbreak control measures → The response was coordinated across the Mano River Union countries → WHO convened the Emergency Committee to assess whether the outbreak constituted a PHEIC → The burden increased to 1779 cases and 961 deaths → A further 221 cases and 106 deaths were reported → WHO maintained its recommendation against general travel or trade restrictions → Surveillance and response budgets were strengthened → The cumulative burden reached 3069 cases and 1552 deaths → More than 40% of cases had occurred during the preceding 21 days → Cases were concentrated in cross-border areas of Guinea, Liberia and Sierra Leone → WHO and its partners strengthened treatment centres, laboratory testing, contact tracing, social mobilisation and safe burial.
4	PEKP-REP-004-RLGE-131	RLGE-131	A patient was referred to a regional hospital and isolated as a suspected viral haemorrhagic fever case → Blood samples were collected and sent to the Uganda Virus Research Institute → RT-PCR confirmed Sudan ebolavirus, and the patient died on the same day → Uganda announced the outbreak → Reported cases reached their peak during 17–23 October 2022 → WHO raised the national risk from high to very high and the regional risk from low to high → New confirmed cases and deaths were reported in Jinja → Kampala subsequently reported a confirmed case → A total of 953 alerts were validated, including 335 meeting the suspected-case definition → The cumulative burden reached 141 confirmed cases and 55 deaths → Nine districts were affected → Contact-follow-up rates were low in Jinja and Masaka → Neighbouring countries reported negative alerts → GOARN received 52 support requests and deployed six experts.
5	PEKP-REP-005-RLGE-132	RLGE-132	Uganda announced an outbreak caused by Sudan ebolavirus → A suspected death was reported → Three new confirmed cases and three deaths occurred among medical staff → WHO raised the national risk from high to very high and the regional risk from low to high → The cumulative burden reached 136 confirmed cases and 53 deaths → Eight districts were affected → Mubende and Kassanda were the most affected districts → An average of 1586 contacts were followed each day → Thirty-four contacts developed symptoms → Of 3867 registered contacts, 2237 completed 21-day follow-up → A total of 2835 alerts were received, of which 94% were investigated within 24 h → A total of 2139 samples were collected.

GOARN – Global Outbreak Alert and Response Network, ID – identifier, PHEIC – Public Health Emergency of International Concern, RT-PCR – reverse transcription polymerase chain reaction, WHO – World Health Organization

## DISCUSSION

### Path results

Analysis of the 47 representative critical paths reveals several recurrent response patterns that event frequency or path length alone cannot explain. First, laboratory confirmation served as a transition from uncertain outbreak signals to an organised response: laboratory-confirmation milestones appeared in 27 paths, and 20 of these were followed by a subsequent non-laboratory intervention, indicating that the critical interval extended from specimen collection and testing to notification, isolation, contact tracing, and field action. Second, healthcare facilities served as both response sites and potential transmission amplifiers. Healthcare-facility-related events appeared in 27 paths, and 18 were followed by later intervention milestones, suggesting that healthcare-worker infection, delayed recognition, or movement between facilities should be treated as sentinel evidence of possible triage or infection-prevention failure. Third, death was not simply the terminal outcome of a path. Death- or burial-related information occurred in 37 paths, and 21 linked a death event to contact tracing or safe-burial action at the same or a later stage, showing that community or unexplained deaths may provide an entry point for reconstructing hidden household, healthcare, or funeral transmission. Fourth, case burden, spatial spread, and international risk represented distinct escalation mechanisms: 41 paths contained burden information, 16 contained spatial-spread signals, and 25 contained international-risk signals, but only seven contained all three. Cross-district cases, referrals, and movement of human remains may therefore connect otherwise separate transmission chains even when the cumulative burden is not yet high. Fifth, international-risk signals were associated mainly with preparedness and coordinated response rather than with border closure; 22 of the 25 paths containing such signals also contained contact tracing, screening-surveillance, or response deployment. Sixth, the paths distinguished the initiation of an intervention from evidence that it had been completed. Contact tracing or screening-surveillance appeared in 38 paths and followed an earlier burden or laboratory-confirmation event in 34, indicating that follow-up completion, negative screening, alert resolution, and the absence of new linked cases can serve as indicators of whether a transmission chain is being closed. Repeated burden updates without corresponding intervention records may therefore represent either a response gap or a documentation gap. Finally, the few paths containing community resistance, insecurity, or operational disruption show that response continuity can itself become a critical-path constraint when access, specimen transport, staffing, or community cooperation fails. Taken together, these findings suggest that the principal vulnerability was not simply the absence of established Ebola-control measures, but the failure to convert epidemiological signals into timely, coordinated, and verifiably completed action.

### Targeted recommendations

In the preceding analysis, we identified and summarised 47 critical paths based on the report content. Building on this, we compared these pathways with WHO recommendations. The advice sections in WHO reports focused on how they described and operationalised recommended measures. These covered key Ebola-control measures such as surveillance and contact tracing, case management, infection prevention and control, laboratory diagnosis, vaccination, safe burial, community engagement, survivor management, preparedness, and travel or trade guidance. Thus, the critical-path findings do not indicate that these measures were missing. Instead, the comparison showed that WHO advice more often described what should be done, but less often specified what events should trigger action, how quickly responses should begin, how responsibilities should be transferred, and how completion should be verified.

Based on this comparison, we found that existing guidance still lacks clarity in ‘action-trigger mechanisms’ and ‘detailed execution processes’. Therefore, supplementary recommendations focus on signal-to-action transition points identified in the critical paths. First, laboratory confirmation should trigger a predefined response package that links result notification with isolation, referral, contact tracing, infection-prevention review, community communication, supply assessment, and, when needed, notification of neighbouring areas. Record key time points, sampling, transport, testing, result notification, and response initiation, to identify delays. Second, healthcare-worker infection or repeated patient visits to different facilities should trigger a time-bound infection-prevention audit covering triage, patient flow, protective equipment, isolation capacity, environmental controls, referral information, and exposure networks. Third, a community or unexplained death should initiate a linked procedure combining retrospective investigation, safe burial, post-mortem sampling where appropriate, and tracing of caregivers and funeral participants. Formal handoffs between burial, surveillance, and tracing teams would help ensure effective follow-up.

In addition, the pathways highlight spatial transmission and cross-border risks, indicating the need for stronger coordination across administrative boundaries. Movement of suspected or confirmed cases, contacts, or human remains across districts or borders should trigger two-way notification, standardised information transfer, protection of transport workers, transfer of contact lists, confirmation of infection control at receiving facilities, and confirmation of arrival and outcome. This targeted coordination should be distinguished from unnecessary travel or trade restrictions. Similarly, high-risk assessments should be directly linked to operational targets such as contact-follow-up coverage, specimen turnaround time, time to isolation, investigation of community deaths, equipment stocks, cross-border notification, and deployment of response teams. In this way, risk assessment becomes an action trigger rather than a descriptive statement.

Finally, analysis of the 47 pathways revealed possible gaps between response implementation and documentation. Repeated updates on cases or deaths without corresponding records of isolation, tracing, laboratory action, vaccination, safe burial, community engagement, or response deployment should trigger alerts indicating potential gaps. These alerts should prompt verification of whether actions were absent, delayed, incomplete, or simply unreported. Because some pathways involved community resistance, insecurity, or operational disruption, outbreak-prone areas should maintain response-continuity measures, including trusted local intermediaries, local teams, alternative transport routes, remote supervision, protected staffing, and minimum capacity for laboratories, beds, transport, and protective equipment.

In summary, these supplementary recommendations address operational gaps not fully covered in WHO guidance. The advice sections only occasionally emphasised real-time monitoring and data-quality requirements, and rarely addressed multi-hazard health-system resilience. Therefore, the critical-path analysis does not replace WHO advice; it extends it by linking established measures with clear triggers, timelines, coordination steps, and evidence of completion.

### Discussion of our method

Most existing research on EVD focuses on clinical diagnosis, descriptive epidemiology, or static knowledge networks, with limited attention to the dynamic event-level evolution of outbreaks [33]. Conventional knowledge graphs typically organise around entities such as pathogens, symptoms, locations, institutions, populations, and dates. Although these graphs integrate heterogeneous epidemiological knowledge, they are less suited to representing how outbreak signals and response measures connect across successive reports. Consequently, they cannot readily show whether laboratory confirmation was followed by intervention, whether community deaths-initiated transmission-chain investigation, or how spatial-spread and international-risk signals were connected to preparedness measures. The recurrent patterns identified in this study broadly align with previous Ebola research but extend it to a different analytical level. Case-based epidemiological and genomic investigations, such as Mbala-Kingebeni *et al.*., have reconstructed individual transmission chains and shown how a single relapse event can lead to geographically dispersed cases [10]. Reviews of previous outbreaks have similarly identified delayed laboratory confirmation, healthcare-facility exposure, unsafe burial practices, and weaknesses in infection prevention and community response as recurrent challenges in Ebola control [34]. Our critical paths reflect these established findings: laboratory confirmation frequently preceded organised intervention, whereas healthcare-worker infections and community deaths often marked points requiring contact tracing, safe burial, or infection-prevention review. This study therefore does not identify new biological transmission mechanisms, but reconstructs how known epidemiological signals connected to subsequent response actions across 346 WHO reports. Compared with the ontology-grounded epidemiological knowledge graph developed by Consoli *et al.*, which primarily structures outbreak entities, attributes, and links to external ontologies [11], our event-centred framework explicitly represents directional relations between events and supports critical-path analysis. This makes cross-report signal-to-response transitions, response interruptions, and possible documentation gaps more visible.

This study addresses this limitation by constructing an event graph from WHO DONs and organising reported events into directional outbreak-evolution chains. The framework connects case detection, laboratory confirmation, case burden, spatial spread, risk assessment, and intervention measures across multiple reports. We evaluated critical paths using six complementary dimensions: case burden, cross-regional or spatial spread, WHO-related international-risk signals, intervention measures, path length, and graph structural importance. We min–max normalised the six dimensions within each event group and summed them with equal weight. The highest-scoring candidate path in each eligible event group was retained as the final critical path. This procedure combines epidemiological evidence with graph structure and prevents any single indicator from determining path selection.

The framework also combines automated processing with manual curation. We used an LLM to transform unstructured WHO reports into event nodes and relations, then consolidated outbreak-level data, removed duplicate events, corrected temporal ordering, and reviewed cycles before generating paths. These procedures reduced fragmentation across successive reports and limited the influence of duplicated events, reversed temporal relations, and cyclic structures on the selected paths.

We generated candidate paths using bounded DFS within each connected event-group component. Although the bounded search strategy improved computational feasibility, critical-path selection in dense event groups may remain sensitive to the predefined search limits and traversal order.

The method provides a structured approach for retrospective infectious-disease intelligence. By identifying laboratory-confirmation-to-response chains, cross-border escalation paths, community-death-to-tracing paths, response gaps, and response-interruption points, it can help locate stages of outbreak evolution that require closer review. Although developed using Ebola reports, the framework may also be applicable to other infectious diseases documented through successive outbreak updates. Further applications should nevertheless adapt the event definitions, relation criteria, and evaluation dimensions to the epidemiological characteristics and reporting practices of the disease under study.

### Limitations

This study has limitations that future research should address. First, this study used only official WHO reports and excluded unofficial community-level surveillance data, which may lead to an incomplete characterisation of hidden community transmission chains. This is in line with the common problem of ‘data reporting bias’ [34] in the study of global infectious disease outbreaks. In some areas, limited monitoring capacity or imperfect reporting mechanisms may underreport outbreaks. Second, the data extraction process omitted some information. When constructing the graph, we did not systematically extract figures and tables embedded in WHO DON reports, which may have omitted quantitative or spatiotemporal information presented only in graphical form. These charts often contain key propagation data, statistical results, and spatiotemporal distribution information. This omission directly leads to the loss of core data, affecting the integrity of graph construction and the accuracy of the analysis results. Third, this study followed a two-stage procedure in which events were first extracted, and relations among them were subsequently classified. Although many event relations were identifiable from the report context, variations in event-boundary definition and the implicit expression of temporal or causal links created uncertainty in relation classification and could result in omissions or misclassification. This problem was particularly evident when distinguishing causality from sequential relations, because descriptive outbreak reports do not always provide sufficient evidence to determine whether a response event was causally triggered by a preceding event or merely occurred later. Accordingly, the causality label in this study represents a text-supported directional dependency rather than a confirmed epidemiological causal effect. Because we retained both Causality and Sequential relations as directional edges during path generation, ambiguity between these labels did not determine whether a relation was eligible for path mining, although it could affect its semantic interpretation and the exact composition of individual paths. Therefore, the extracted critical paths should be interpreted as directional associations among reported outbreak and response events rather than as verified epidemiological causal chains. Future research should combine improved relation-classification models with expert adjudication to reduce event-boundary errors and strengthen the distinction between causal and sequential relations. The Event type-candidate relation matrix used to guide relation classification was a heuristic constraint rather than an independently validated epidemiological causation framework. We developed it from recurrent event-type co-occurrence patterns in WHO outbreak reports and domain-informed expectations about plausible links among outbreak detection, laboratory confirmation, spatial spread, risk assessment, and intervention measures. Its purpose was to restrict implausible model outputs and support consistent relation classification, rather than to provide definitive evidence of causation. The matrix should therefore be further refined and validated using expert-reviewed epidemiological evidence and broader outbreak data sets.

## CONCLUSIONS

We proposed an event-graph-based framework to address the limited ability of conventional descriptive summaries and entity-centred knowledge graphs to reconstruct how Ebola outbreaks evolve across successive reports. By linking fragmented outbreak signals, transmission-related events, risk assessments, and response actions into ordered event chains, the framework provides a more structured representation of how reported outbreaks and public-health responses evolve over time, rather than relying on static aggregation of information. The critical-path evaluation further distinguishes epidemiologically meaningful event chains by disease burden, spatial spread, international risk, intervention measures, path length, and structural importance.

The findings indicate that an effective Ebola response depends not only on the availability of established control measures, but also on activating these measures at appropriate transition points. Laboratory confirmation, healthcare-worker infection, community deaths, spatial spread, and elevated risk assessments emerged as important signals requiring coordinated follow-up. Comparison with WHO advice showed that existing recommendations generally cover the principal Ebola-control measures, but provide less consistent detail on when specific signals should trigger action, how rapidly responses should begin, how responsibilities should be transferred across teams or administrative boundaries, and how implementation should be verified. Accordingly, this study recommends linking key outbreak signals to predefined, time-bound, auditable response procedures; strengthening coordination among surveillance, laboratory, clinical, burial, contact-tracing, and cross-border teams; and establishing alerts for potential response or documentation gaps.

Overall, the event-graph framework complements rather than replaces WHO guidance, real-time surveillance, or formal epidemiological causal inference. Its main contribution is transforming fragmented outbreak reports into interpretable response trajectories and identifying operational points at which established control measures should be initiated, coordinated, and monitored. Future research should incorporate broader surveillance and graphical sources, strengthen expert validation, and evaluate the framework's robustness and generalisability across different outbreaks and reporting systems.

## Additional material


Online Supplementary Document

## Data Availability

**Data availability:** The data sets used and analysed in this study are available from the corresponding author upon reasonable request.
