# Supplementary material for: Constructing event graph and mining critical paths of Ebola virus disease: a public health emergency analysis based on WHO disease outbreak news
Source: J Glob Health. 2026 Oct 30;16:04341. doi: 10.7189/jogh.16.04341 (PMC13721838; doi:10.7189/jogh.16.04341)
Supplement: Supplementary file 1 [file jogh-16-04341-s001.pdf]

Supplement to: Ni Z, Sun Y, Feng G, Wei J. Constructing event graph and mining critical paths of Ebola virus disease: a public health emergency analysis based on WHO Disease Outbreak News. J Glob Health. 2026;16:04341.

Contents

Appendix S1. Model settings

Appendix S2. Prompt template for event extraction

Appendix S3. Prompt template for event relation extraction

Appendix S4. Annotator information

Table S1. Representative extracted events

Table S2. Representative event-relation results

Checklist S1. Completed GAMER checklist

Appendix S1. Model settings

The event extraction and event-relation extraction procedures were assisted by DeepSeek-R1 through the official DeepSeek API (API model: deepseek-reasoner). The model was used from 2025-07-11 to 2025-08-20. No separately identifiable model version or snapshot was available for these API calls. The main model settings were as follows:

Provider: DeepSeek official API

Model: DeepSeek-R1 (API model: deepseek-reasoner)

Use period: 2025-07-11 to 2025-08-20

Maximum output tokens: 32,000

Temperature: 0.3

Top-p: 0.9

The model was instructed to return structured outputs only. Explanations and intermediate reasoning were not included in the final structured outputs. Prompt templates are provided in Appendices S2 and S3. Only representative outputs were retained and are provided in Tables S1 and S2; the complete unedited model responses were not preserved.

DeepSeek-R1 was also used to improve the readability and language expression of the manuscript. The authors reviewed and edited the AI-assisted content as necessary and take full responsibility for the content of the publication.

All data submitted to the model were obtained from publicly available WHO Disease Outbreak News. No personal, confidential, or unpublished data were processed.

Appendix S2. Prompt template for event extraction

The event extraction prompt was designed to standardize unstructured WHO Disease Outbreak News reports into temporally ordered public health event statements.

System role:

You are an epidemic text normalization expert. You must strictly follow the user-provided steps and examples to transform unstructured epidemic text into chronologically clear and consistently formatted structured content. Only output the normalized result. Do not include analysis, explanation, or reasoning process.

Task steps:

Step 1: Text cleaning.

Remove navigation text and repeated boilerplate information, such as “See all DONs related to this event”. Remove repeated statements while retaining key information at its first occurrence.

Step 2: Temporal ordering.

Extract time markers, such as “2022-11-27”, and organize events according to chronological order. The preferred output format is “[time] event”.

Step 3: Entity Standardization.

Standardize institutional names, disease names, virus subtype names, and country or regional names. For example, “WHO” should be normalized to “World Health Organization”, “SUDV” to “Sudan ebolavirus”, and country or regional names should be made consistent.

Step 4: Numeric and date standardization.

Normalize dates into a consistent format, preferably “YYYY-MM-DD” when available. Quantitative information should be clearly retained and labelled, such as confirmed cases, deaths, case fatality rate, suspected cases, contacts, vaccination counts, and follow-up rates.

Step 5: Event Element Extraction

Extract all event components as defined by the multi-tuple event representation to the maximum possible degree. The extraction scope covers the temporal attribute , spatial attribute , participant set , core action description , and supplementary event attributes (event type, polarity, tense, etc.). Given the ambiguous and incomplete information carried by unstructured epidemic text, tuple elements absent from the original text do not need to be supplemented. Only recognizable semantic elements are kept to ensure event distinguishability and enable subsequent event relation modeling.

Example input:

WHO Disease Outbreak News text containing sections such as Description of the situation, Public health response, WHO risk assessment, and WHO advice.

Example output format:

> [2022-09-20] Uganda declared an outbreak of Ebola disease caused by Sudan ebolavirus;
>
> [2022-11-04] The World Health Organization revised the rapid risk assessment: the national risk level in Uganda increased from high to very high, the regional risk level increased from low to high, and the global risk level remained low;
>
> [2022-12-05] As of this date, 142 confirmed cases and 55 deaths were reported, with a case fatality rate of 39%;
>
> [2022-12-08] Candidate vaccines arrived in Uganda for clinical trials.

Appendix S3. Prompt template for event relation extraction

The event relation extraction prompt was designed to classify the relationship between two extracted public health events. The output relation types were limited to Causality, Sequential, Parallel, and Unrelated.

System role:

You are a professional event relation analysis assistant. Analyze the relationship between two epidemic-related events according to the following steps.

Step 1: Identify the event type.

First determine which event category each event belongs to. The available event categories are: early epidemic event, epidemic outbreak event, case report event, epidemic response event, medical intervention event, and epidemic termination event.

Examples:

– “The first confirmed case appeared and the local area entered epidemic alert” → early epidemic event.

– “Daily confirmed cases exceeded 100 and community transmission began” → epidemic outbreak event.

– “A hospital reported three unexplained pneumonia cases” → case report event.

Step 2: Refer to candidate event-type relations.

Use the empirical event type–candidate relation matrix as an initial reference only. The matrix provides candidate relation types between ordered event-type pairs, but it does not determine the final relation by itself.

Candidate relation guidance:

The empirical event type–candidate relation matrix used to guide relation extraction is provided as Table 1 in the main manuscript.

Step 3: Analyze report-level association.

If the two events are from the same WHO report or from successive reports describing the same outbreak, they are considered more likely to be related. If the two events do not share report context, outbreak context, time continuity, or geographical relevance, treat the relation cautiously.

Step 4: Output the relation label.

The final relation type must be limited to one of the following: Causality, Sequential, Parallel, or Unrelated. Only return the relation type. Do not add an explanation.

Appendix S4. Annotator information

Annotator 1: Ziran Ni is a student at the School of Management, Nanjing University of Posts and Telecommunications. His research interest is library and information management. He participated in event identification and event-relation annotation.

Annotator 2: Jianxiang Wei is a professor and doctoral supervisor at Nanjing University of Posts and Telecommunications. His research interests include intelligent information processing, data mining, and knowledge discovery. He also participated directly in event identification and event-relation annotation.

Both annotators followed the same event definitions, relation-label criteria, and annotation guidelines. Differences between their annotation judgments were discussed and resolved before the final validation results were calculated.

Table S1. Representative extracted events

Number	Event content	Event date or period	Source report
1	Uganda announced an outbreak of Ebola disease caused by Sudan virus.	2022-09-20	Ebola disease caused by Sudan ebolavirus – Uganda [2022-12-08]
2	WHO organized expert consultations to identify candidate treatments and vaccines and to develop clinical-trial protocols. Experts recommended three candidate vaccines for ring-vaccination trials: VSV-SUDV (Merck/IAVI), ChAd3-SUDV (Sabin Vaccine Institute), and biEBOV (Oxford University/Jenner Institute).	2022-10 to 2022-11	Ebola disease caused by Sudan ebolavirus – Uganda [2022-12-08]
3	WHO revised its rapid risk assessment: the national risk for Uganda increased from high to very high, the regional risk increased from low to high, and the global risk remained low.	2022-11-04	Ebola disease caused by Sudan ebolavirus – Uganda [2022-12-08]
4	The preceding Disease Outbreak News update was published.	2022-11-24	Ebola disease caused by Sudan ebolavirus – Uganda [2022-12-08]
5	A new confirmed case was reported in Kassanda District: a stillborn baby delivered at 28 weeks of gestation to a mother who had recovered after infection with Sudan virus during pregnancy.	2022-11-27	Ebola disease caused by Sudan ebolavirus – Uganda [2022-12-08]

Dates are presented in ISO 8601 format at the precision supported by the source: exact dates use YYYY-MM-DD, whereas the month-level period uses YYYY-MM. Abbreviations: biEBOV – bivalent Ebola virus vaccine; ChAd3-SUDV – chimpanzee adenovirus type 3 vaccine against Sudan virus; IAVI – International AIDS Vaccine Initiative; SUDV – Sudan virus; VSV-SUDV – vesicular stomatitis virus-vectored vaccine against Sudan virus; WHO – World Health Organization.

Table S2. Representative event-relation results

Event 1	Event 2	Relation
Uganda announced an outbreak of Ebola caused by Sudan virus (2022-09-20).	WHO revised the rapid risk assessment: Uganda's national risk increased from high to very high, the regional risk increased from low to high, and the global risk remained low (2022-11-04).	Sequential
WHO organized expert consultations to identify candidate treatments and vaccines and to develop clinical-trial protocols. Experts recommended VSV-SUDV, ChAd3-SUDV, and biEBOV for ring-vaccination trials (2022-10 to 2022-11).	WHO revised the rapid risk assessment: Uganda's national risk increased from high to very high, the regional risk increased from low to high, and the global risk remained low (2022-11-04).	Parallel
Uganda announced an outbreak of Ebola caused by Sudan virus (2022-09-20).	The preceding Disease Outbreak News update was published (2022-11-24).	Sequential
Uganda announced an outbreak of Ebola caused by Sudan virus (2022-09-20).	WHO organized expert consultations to identify candidate treatments and vaccines and to develop clinical-trial protocols. Experts recommended VSV-SUDV, ChAd3-SUDV, and biEBOV for ring-vaccination trials (2022-10 to 2022-11).	Sequential

Abbreviations are defined in the footnote to Table S1. Month-level periods use YYYY-MM because exact calendar dates were not stated in the source report.

Checklist S1. Completed GAMER checklist

This checklist reports the use of generative artificial intelligence in the study and manuscript according to the GAMER Statement.

No.	GAMER reporting item	Reported	Where reported and completed response
1	Declaration of generative artificial intelligence (GAI) use	Yes	DeepSeek-R1 was used for event extraction, event-relation extraction, and language editing. Locations: main manuscript, Methods—Information extraction and the GAI-use disclosure; Online Supplementary Document, Appendices S1–S3.
2	Tool specifications, version information, and use dates	Yes	Official DeepSeek API; DeepSeek-R1; API model identifier: deepseek-reasoner; no separately identifiable version or snapshot was available; used from 2025-07-11 to 2025-08-20. Location: Appendix S1.
3	Prompting technique and availability of unedited responses	No*	Structured prompts used role constraints, stepwise reasoning instructions, predefined output formats, and few-shot examples. Prompt templates are provided in Appendices S2 and S3, and representative outputs are provided in Tables S1 and S2. The complete unedited responses were not preserved; therefore, this item is not fully satisfied.
4	Development or fine-tuning of a new GAI model	N/A	No new GAI model was developed, trained, or fine-tuned. DeepSeek-R1 was accessed through the official API.
5	Role of GAI tools in every study phase in which they were used	Yes	DeepSeek-R1 generated structured event records, classified event-pair relations, and assisted with language editing. It did not perform the independent expert validation or determine the final interpretation. Locations: main manuscript, Methods—Information extraction and Validation of model-based information extraction; Appendices S1–S4.

*Prompting was reported, but the complete unedited responses were not preserved.

Checklist S1. Completed GAMER checklist (continued)

No.	GAMER reporting item	Reported	Where reported and completed response
6	Specific manuscript sections or paragraphs assisted by GAI	Yes	DeepSeek-R1 contributed to the event and event-relation extraction procedures reported in the Methods and to the results derived from those structured outputs. It was also used to improve readability and language expression throughout the manuscript. The authors reviewed and edited all AI-assisted text.
7	Verification and, where necessary, modification of GAI-generated content	Yes	Two annotators independently validated sampled events and event-pair relations; disagreements were resolved through discussion. Model outputs were compared with the agreed reference annotations. Outbreak-level consolidation, duplicate removal, relation correction, and cycle removal were performed before critical-path mining. Locations: main manuscript, Methods—Validation of model-based information extraction and Table 2; Appendix S4.
8	Protection of data privacy and confidentiality	Yes	Only publicly available WHO Disease Outbreak News text was submitted to the model. No personal, confidential, unpublished, or otherwise restricted data were processed. Location: Appendix S1.
9	Possible influence of GAI use on interpretation, accuracy, or conclusions	Yes	The GAI-derived structured outputs formed part of the event-graph input and could introduce omissions, boundary inconsistencies, or relation misclassification. Manual validation and downstream corrections were used to reduce these risks, and the findings were interpreted as text-supported directional associations rather than confirmed epidemiological causal effects. The authors take full responsibility for the interpretation and conclusions. Locations: main manuscript, Results, Discussion, and Limitations.

Abbreviations: AI – artificial intelligence; GAI – generative artificial intelligence; GAMER – Generative Artificial intelligence tools in MEdical Research; N/A – not applicable.

Checklist source: Luo X, Tham YC, Giuffrè M, et al. Reporting guideline for the use of Generative Artificial intelligence tools in MEdical Research: the GAMER Statement. BMJ Evid Based Med. 2025;30:390–400. doi:10.1136/bmjebm-2025-113825.
